# Supplementary material for: The Association of Social Support and Loneliness with Symptoms of Depression, Anxiety, and Posttraumatic Stress during the COVID-19 Pandemic: A Meta-Analysis
Source: Int J Environ Res Public Health. 2023 Feb 4;20(4):2765. doi: 10.3390/ijerph20042765 (PMC9957328; doi:10.3390/ijerph20042765)
Supplement: Supplementary file 1 [file ijerph-20-02765-s001.zip › ijerph-2189661-supplementary.pdf]

Supplementary Material File S1

Table S1. Overview of the search terms used in each database.

**Pubmed**		**ScienceDirect[a]**		**Web of Science[b]**	
Search terms	Boolean operators	Search terms	Boolean operators	Search terms	Boolean operators
Social support [Mesh]	OR	Social support [kwd]	OR	Social support [kwd]	OR
Social networks [kwd]	OR	Social networks [kwd]	OR	Social networks [kwd]	OR
Social connectedness [kwd]	OR	Social relationships [kwd]	OR	Social connectedness [kwd]	OR
Social relationships [kwd]	OR	Social isolation [kwd]	OR	Social relationships [kwd]	OR
Social isolation [Mesh]	OR	Loneliness [kwd]	AND	Social isolation [kwd]	OR
Loneliness [Mesh]	AND	Mental health [kwd]	OR	Loneliness [kwd]	AND
Mental health [Mesh]	OR	Mental disorders [kwd]	AND	Mental health [kwd]	OR
Mental disorders [Mesh]	OR	COVID-19 [kwd]	OR	Mental disorders [kwd]	OR
Anxiety disorders [Mesh]	OR	Lockdown [kwd]	AND	Anxiety disorders [kwd]	OR
Depressive disorders [Mesh]	OR			Depressive disorders [kwd]	OR
Depression [Mesh]	OR			Depression [kwd]	OR
Anxiety [Mesh]	OR			Anxiety [kwd]	OR
Stress disorders, post-traumatic [Mesh]	OR			Post-traumatic [kwd]	OR
stress disorder [kwd]	AND			stress disorder [kwd]	AND
COVID-19 [Mesh]	OR			COVID-19 [kwd]	OR
Lockdown [kwd]	OR			Lockdown [kwd]	OR
Quarantine [kwd]	OR			Quarantine [kwd]	OR
Pandemic [kwd]	AND			Pandemic [kwd]	AND
"2020/01/01" [Date - Publication] : "2022/10/03" [Date - Publication]		Year "2020-2022"		DOP=(2020-01-01/2022-10-03)	
**Hits:**	**835**	**Hits:**	**532**	**Hits:**	**2792**

Note: [a]ScienceDirect search was filtered by 'Article type' selecting 'Research articles'; [b]Web of science search keywords used the advanced search field tag AB, which searches the Abstract field within a record, and the search was filtered by 'Type of document' selecting 'Article'.

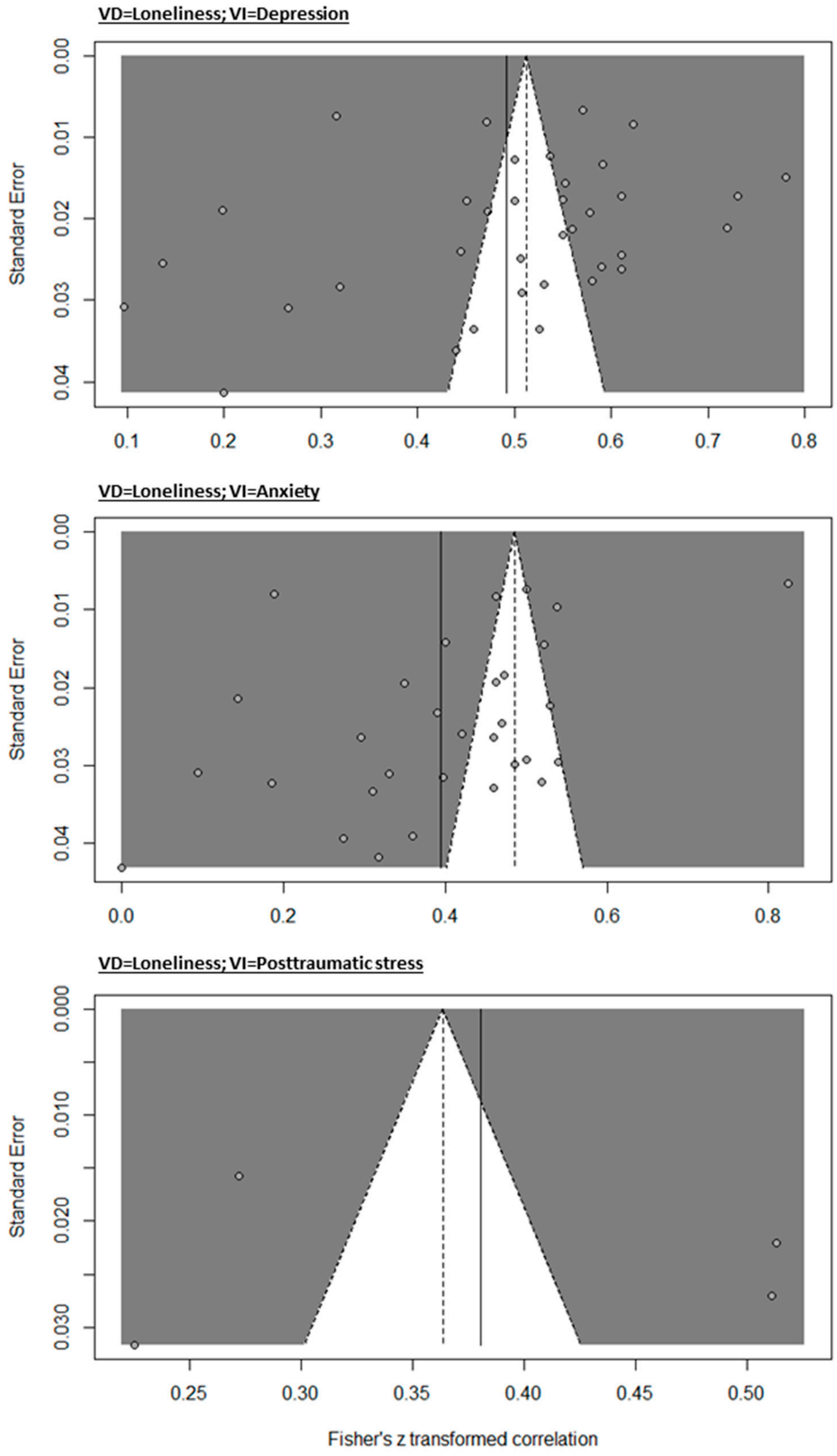

Figure S1. Funnel plots representing the standard error by Fisher's Z for the loneliness' effect sizes showing the symmetry of the data in relation to publication bias.

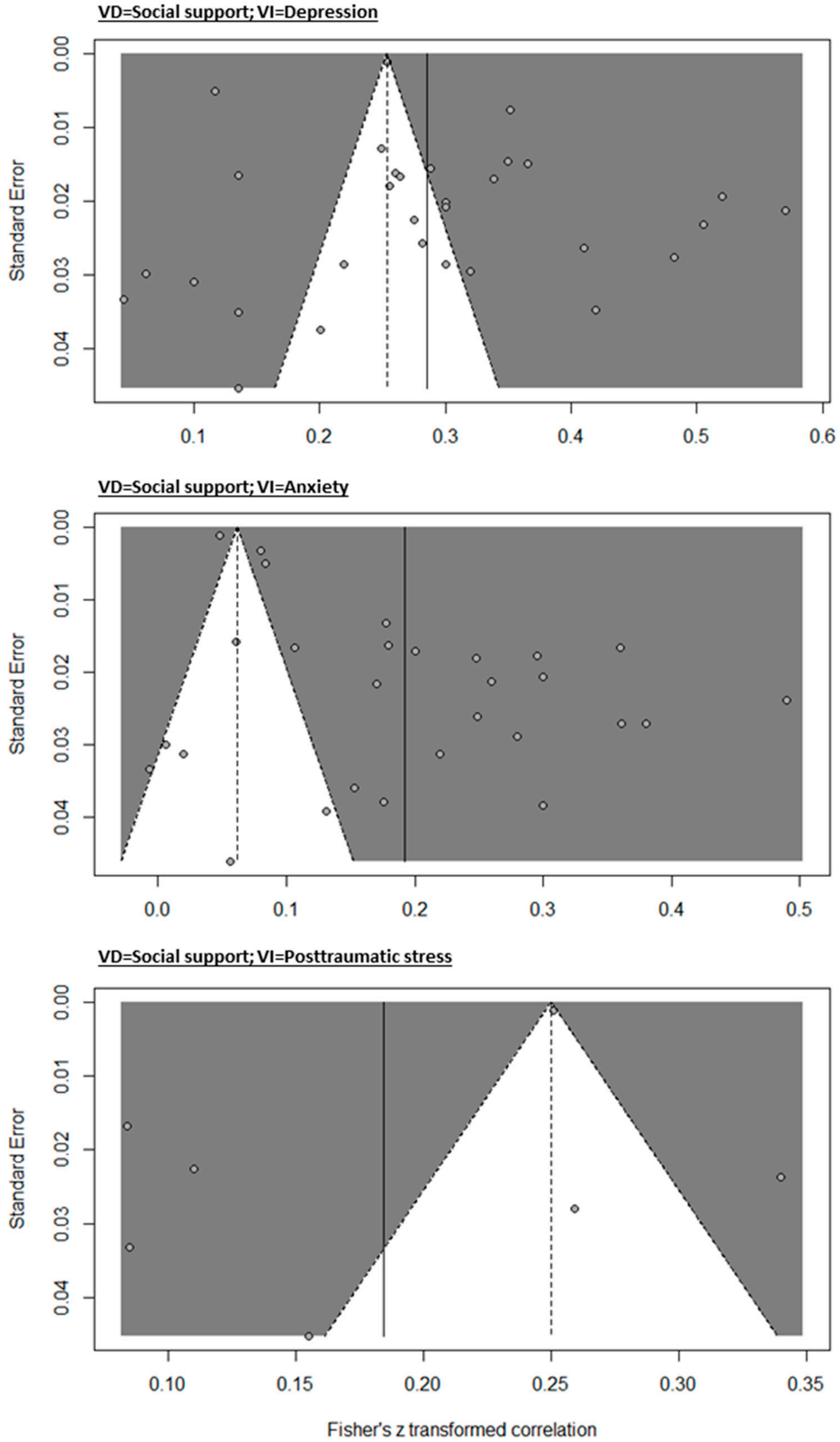

Figure S2. Funnel plots representing the standard error by Fisher's Z for the social support effect sizes showing the symmetry of the data in relation to publication bias.

Supplementary Material File S2

Adapted version of the Newcastle Ottawa Scale (NOS).

Newcastle-Ottawa Scale adapted for cross-sectional studies

Selection (maximum 5 stars):

1. Representativeness of the sample (max. 1 *):
   a. Truly representative of the average in the target population. * (all subjects or random sampling)
   b. Somewhat representative of the average in the target group. * (non-random sampling)
   c. Selected group of users/convenience sample.
   d. No description of the sampling strategy.
2. Sample size (max. 1 *):
   a. Justified and satisfactory (including sample size calculation). *
   b. Not justified or no information provided.
3. Non-respondents (max. 1 *):
   a. Score if one of the following items were fulfilled: *
      ✓ Comparability between respondents and non-respondents' characteristics is established, and the response rate is satisfactory.
      ✓ Response rate=100%.
      ✓ The study employed multiple imputation approach to account for the uncertainty in missing responses.
   b. The response rate is unsatisfactory, or the comparability between respondents and non-respondents is unsatisfactory.
   c. No description of the response rate or the characteristics of the responders and the non-responders.
4. Ascertainment of the exposure [social support or/and loneliness] (max. 2 *):
   a. Validated measurement tool for the study setting and language. **
   b. Measurement tool not validated for the specific study setting and language, but the tool is validated in other populations. *
   c. Non-validated measurement tool or no description of the measurement tool.

Comparability (maximum 2 stars):

1. Comparability of subjects in different outcome groups on the basis of design or analysis. Confounding factors controlled.
   a. The study controls for age and sex *
   b. The study controls for other relevant factors (economic, education, marital status, pre-pandemic mental disorders) *
   c. Data/results not adjusted for all relevant confounders/risk factors/information not provided.

Outcome (maximum 3 stars):

1. Assessment of outcome [depression, anxiety, or PTSD symptoms] (max. 2 *):
   a. Validated measurement tool for the study setting and language. **

   b. Measurement tool not validated for the specific study setting and language, but the tool is validated in other populations. *
   c. Non-validated measurement tool or no description of the measurement tool.
2. Statistical test (max. 1 *):
   a. The statistical test used to analyse the data is clearly described and appropriate, and the measurement of the association is presented, including confidence intervals and the probability level (p-value). *
   b. Statistical test not appropriate, not described or incomplete.

Newcastle-Ottawa Scale adapted for longitudinal studies

Selection (maximum 5 stars):

1. Representativeness of the sample (max. 1 *):
   a. Truly representative of the average in the target population. * (all subjects or random sampling)
   b. Somewhat representative of the average in the target group. * (non-random sampling)
   c. Selected group of users/convenience sample.
   d. No description of the sampling strategy.
2. Sample size (max. 1 *):
   a. Justified and satisfactory (including sample size calculation). *
   b. Not justified or no information provided
3. Non-respondents (max. 1 *):
   a. Score if one of the following items were fulfilled: *
      - ✓ Comparability between respondents and non-respondents' at follow-up is established, and the response rate is satisfactory.
      - ✓ Response rate=100%.
      - ✓ The study employed multiple imputation approach to account for the uncertainty in missing responses.
   b. The response rate at baseline is unsatisfactory, or the comparability between respondents and non-respondents is unsatisfactory or the variables assessment is different between waves.
   c. No description of the response rate or the characteristics of the responders and the non-responders.
4. Ascertainment of the exposure [social support or/and loneliness] (max. 2 *):
   a. Validated measurement tool for the study setting and language. **
   b. Measurement tool not validated for the specific study setting and language, but the tool is validated in other populations. *
   c. Non-validated measurement tool or no description of the measurement tool.

Comparability (maximum 2 stars):

1. Comparability of subjects in different outcome groups on the basis of design or analysis. Confounding factors controlled.
   a. The study controls for age and sex *
   b. The study controls for other relevant factors (economic, education, marital status, pre-pandemic mental disorders) *
   c. Data/results not adjusted for all relevant confounders/risk factors/information not provided.

Outcome (maximum 3 stars):

1. Assessment of outcome [depression, anxiety, or PTSD symptoms] (max. 2 *):
    a. Validated measurement tool for the study setting and language. **
    b. Measurement tool not validated for the specific study setting and language, but the tool is validated in other populations. *
    c. Non-validated measurement tool or no description of the measurement tool.
2. Statistical test (max. 1 *):
    a. Complete follow-up, all subjects accounted for. *
    b. Subjects lost to follow-up unlikely to introduce bias: number lost less than or equal to 20% or description of those lost suggested no different from those followed. *
    c. Follow-up rate less than 80% and no description of those lost.
    d. No statement.

Thresholds for converting the Newcastle-Ottawa scales to AHRQ standards (good, fair, and poor):

- **Good quality**: 3 or more stars in selection domain AND 1 or 2 stars in comparability domain AND 2 or 3 stars in outcome/exposure domain
- **Fair quality**: 2 stars in selection domain AND 1 or 2 stars in comparability domain AND 2 or 3 stars in outcome/exposure domain
- **Poor quality**: 0 or 1 star in selection domain OR 0 stars in comparability domain OR 0 or 1 stars in outcome/exposure domain
